# Inflammation and glucose homeostasis are associated with specific structural features among adults without knee osteoarthritis: a cross-sectional study from the osteoarthritis initiative

**DOI:** 10.1186/s12891-017-1921-6

**Published:** 2018-01-05

**Authors:** Alina C. Stout, Mary F. Barbe, Charles B. Eaton, Mamta Amin, Fatimah Al-Eid, Lori Lyn Price, Bing Lu, Grace H. Lo, Ming Zhang, Jincheng Pang, Timothy E. McAlindon, Jeffrey B. Driban

**Affiliations:** 1 Division of Rheumatology, Tufts Medical Center, 800 Washington Street, Box #406, Boston, MA 02111 USA; 2 Department of Anatomy and Cell Biology, Temple University School of Medicine, 3500 North Broad Street, Philadelphia, PA 19140 USA; 3 Center for Primary Care and Prevention, Alpert Medical School of Brown University, 111 Brewster Street, Pawtucket, RI 02860 USA; 4 The Institute for Clinical Research and Health Policy Studies, Tufts Medical Center, 800 Washington Street, Box #63, Boston, MA 02111 USA; 5 Tufts Clinical and Translational Science Institute, Tufts University, 800 Washington Street, Box #63, Boston, MA 02111 USA; 6 Brigham & Women’s Hospital and Harvard Medical School, 75 Francis Street PBB-B3, Boston, MA 02115 USA; 7 Medical Care Line and Research Care Line, Houston Health Services Research and Development (HSR&D) Center of Excellence Michael E. DeBakey VAMC, 2002 Holcombe Blvd, Houston, TX 77030 USA; 8 Section of Immunology, Allergy, and Rheumatology, Baylor College of Medicine, 1 Baylor Plaza, BCM-285, Houston, TX 77030 USA; 9 Department of Electrical Engineering, Tufts University, 161 College Avenue, Medford, MA 02155 USA

**Keywords:** Bone marrow lesions, Effusion, Magnetic resonance imaging, Osteoarthritis

## Abstract

**Background:**

Greater age and body mass index are strong risk factors for osteoarthritis (OA). Older and overweight individuals may be more susceptible to OA because these factors alter tissue turnover in menisci, articular cartilage, and bone via altered glucose homeostasis and inflammation. Understanding the role of inflammation and glucose homeostasis on structural features of early-stage OA may help identify therapeutic targets to delay or prevent the onset of OA among subsets of adults with these features. We examined if serum concentrations of glucose homeostasis (glucose, glycated serum protein [GSP]) or inflammation (C-reactive protein [CRP]) were associated with prevalent knee bone marrow lesions (BMLs) or effusion among adults without knee OA.

**Methods:**

We conducted a cross-sectional study using baseline data from the Osteoarthritis Initiative. We selected participants who had no radiographic knee OA but were at high risk for knee OA. Blinded staff conducted assays for CRP, GSP, and glucose. Readers segmented BML volume and effusion using semi-automated programs. Our outcomes were prevalent BML (knee with a BML volume > 1 cm^3^) and effusion (knee with an effusion volume > 7.5 cm^3^). We used logistic regression models with CRP, GSP, or glucose concentrations as the predictors. We adjusted for age, sex, body mass index (BMI), and Physical Activity Scale for the Elderly (PASE) scores.

**Results:**

We included 343 participants: mean age = 59 ± 9 years, BMI = 27.9 ± 4.5 kg/m^2^, PASE score = 171 ± 82, and 64% female. Only CRP was associated with BML prevalence (odds ratio [OR] = 1.43, 95% confidence interval [CI] = 1.09 to 1.87). For effusion, we found an interaction between BMI and CRP: only among adults with a BMI <25 kg/m^2^ was there a significant trend towards a positive association between CRP and effusion (OR = 1.40, 95% CI = 1.00 to 1.97). We detected a U-shaped relationship between GSP and effusion prevalence. Fasting glucose levels were not significantly associated with the presence of baseline effusion or BML.

**Conclusions:**

Among individuals without knee OA, CRP may be related to the presence of BMLs and effusion among normal weight individuals. Abnormal GSP may be associated with effusion. Future studies should explore whether inflammation and glucose homeostasis are predictive of symptomatic knee OA.

## Background

Greater age and body mass index are strong risk factors for osteoarthritis (OA) [1–4]. Older and overweight individuals may be more susceptible to OA because these factors alter tissue turnover in menisci, articular cartilage, and bone via altered glucose homeostasis [5–9] and inflammation [8, 10–14]. Individuals with osteoarthritis (OA) have greater systemic inflammation [15] and impaired glucose homeostasis [16]. However, it remains unclear if systemic inflammation and glucose homeostasis relate to specific structural features of early-stage OA, namely bone marrow lesions (BMLs) and effusion. BMLs and effusion are common features of OA that are associated with incidence of disease and symptoms [17–20]. Specifically, BMLs may reflect changes in the subchondral bone (e.g., fibrosis, edema, necrosis, abnormal trabeculae) that may predispose the joint to articular cartilage loss and greater knee pain [17]. Meanwhile, effusion and synovitis are linked to incident radiographic knee OA up to 2 years prior to diagnosis [19]. BMLs and effusion are particularly interesting because they can change size in less than 12 weeks [21, 22]. Furthermore, clinical interventions may reduce BML size [23, 24] and effusion volume [22].

Understanding the role of inflammation and glucose homeostasis on structural features of early-stage OA may help identify therapeutic targets to delay or prevent the onset of OA among subsets of adults with these features. C-reactive protein (CRP) is a common clinical biomarker used to test systemic inflammation [25]. Individuals with greater CRP concentration are more likely to have greater OA-related symptoms [26] and develop incident radiographic OA [27]. In relation to specific structural features of knee OA, one study found that CRP may be associated with BMLs among adults with knee OA [28] but other investigators reported null findings among individuals with and without OA [29]. Hence, we evaluated CRP because it may play a role in OA pathophysiology, the biomarker is clinically available, and prior studies assessed CRP in relation to OA.

Despite evidence that metabolic syndrome and diabetes may be associated with OA there is limited evidence about the role of glucose homeostasis on BMLs or effusion [30]. To assess altered glucose homeostasis, we assessed glucose concentrations, which offers an assessment of glucose levels at the time of the study visit, and glycated serum protein (GSP), which provides a stable marker of glucose homeostasis over the 2 to 3 weeks prior to the study visit. It would be valuable to further explore the association between inflammation and glucose homeostasis among individuals without knee OA.

We sought to expand prior research by examining only individuals without radiographic knee OA. We assessed CRP and glucose concentrations, which had been tested in prior studies, and GSP, which provides a stable indicator of glucose homeostasis. The purpose of this study was to determine if serum concentrations of impaired glucose homeostasis (glucose or GSP) or systemic inflammation (CRP) were associated with the prevalence of knee BMLs or effusion. We focused on serum concentrations of these measures because serum would be easier to collect in most clinical settings than other samples (e.g., synovial fluid). Our long-term goal is to understand the relationship between glucose and/or inflammatory impairment and BMLs or effusion. This study represents a key early step to understanding whether systemic inflammation or irregular glucose homeostasis predisposes a knee to BMLs or effusion or whether BMLs and effusion lead to systemic inflammation and glucose irregularity.

## Methods

The Osteoarthritis Initiative (OAI) is a longitudinal, multi-center, observational study of 4796 individuals with or at risk for knee OA at four sites in the United States: Memorial Hospital of Rhode Island, The Ohio State University, University of Maryland and Johns Hopkins University, and the University of Pittsburgh. The study staff enrolled men and women (45 to 79 years of age) between February 2004 and May 2006. Descriptions of the eligibility criteria and the OAI protocol are publicly available on the OAI website [31]. The OAI study was approved by institutional review boards at each OAI clinical site and the coordinating center: Memorial Hospital of Rhode Island Institutional Review Board, The Ohio State University’s Biomedical Sciences Institutional Review Board, University of Pittsburgh Institutional Review Board, University of Maryland Baltimore – Institutional Review Board, and Committee on Human Research at University of California, San Francisco. All participants provided informed consent.

**Fig. 1:**
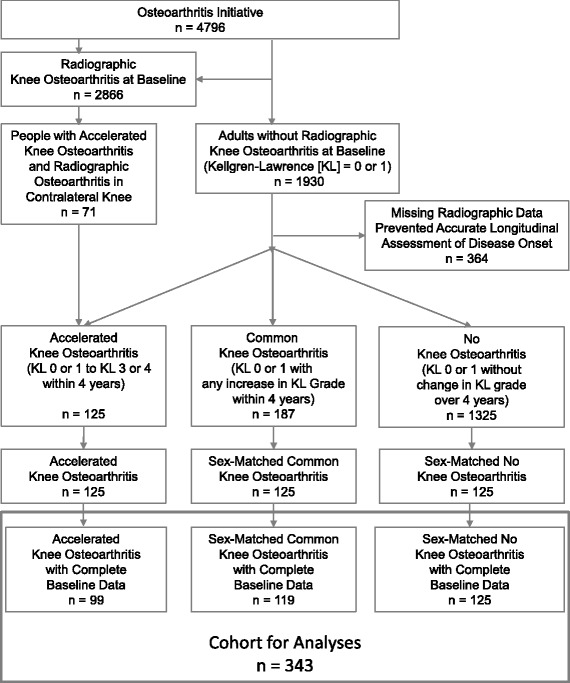
Flow chart of eligibility for participants in analyses

To determine if serum concentrations of impaired glucose homeostasis (glucose or GSP) or systemic inflammation (CRP) were associated with the prevalence of knee BMLs or effusion, we conducted a secondary analysis among a well characterized case-control sample that included three groups (*n* = 125/group): development of accelerated knee OA (case), common knee OA, or no knee OA (Fig. 1) [32–34]. One-hundred and twenty-five knees had no radiographic OA (Kellgren-Lawrence [KL] grade < 2) at the OAI baseline but then developed advanced-stage knee OA (KL grade 3 or 4) within 4 years. Knees from the other 2 groups were randomly matched by sex to the cases in a 1:1 ratio. Common knee OA was defined as no radiographic knee OA at baseline and progressed in KL grade ≥ 1 but did not reach advanced-stage knee OA over the course of 4 years. No knee OA was defined as no radiographic knee OA at baseline and had no change in KL grade between the baseline visit and 4 years thereafter. From the 3 groups (*n* = 375), we included 343 individuals who had sagittal intermediate-weighted, turbo spin echo, fat-suppressed magnetic resonance (MR) images and fasting serum data available at baseline (accelerated knee OA: *n* = 99, common knee OA: *n* = 119, no knee OA: *n* = 125). This sample of participants offered us the advantage of a rich dataset and enabled us to ensure an adequate sample size of knees had baseline BMLs and effusion.

We included one knee per person. Among those who progressed over the subsequent 4 years, we included the knee with the greatest radiographic progression. For individuals with no radiographic progression, we selected a knee to match one person of the same sex who developed advanced-stage radiographic knee OA.

### MR image acquisition

To assess BMLs and knee effusion volume we used sagittal intermediate-weighted, turbo spin echo, fat-suppressed MR images which were acquired at the OAI baseline visit (field of view = 160 mm, slice thickness = 3 mm, skip = 0 mm, flip angle = 180 degrees, echo time = 30 ms, recovery time = 3200 ms, 313 × 448 matrix (interpolated to 512 × 512), phase encode superior/inferior, × resolution = 0.357 mm, and y resolution = 0.511 mm) [35]. All MR sequences were taken using one of four identical Siemens Trio 3-Tesla MR systems and a USA Instruments quadrature transmit-receive knee coil at one of the four OAI clinical sites. The OAI protocol for MR imaging is available on the OAI website [31].

### BML measurements

A single reader (ACS) used a semi-automated program, which we described previously [36–38], to measure BMLs in the distal femur and proximal tibia on sagittal intermediate-weighted, turbo spin echo, fat-suppressed MR images. First, the reader identified the first and last slices in the MR sequence in which the tibia or femur are present. Following this, the reader marked several points along the articular surface of both the tibia and femur to define the crude boundaries of each bone. To mark the border furthest from the articular surface the reader marked the bone just prior to the epiphyseal line. Once the reader completed the manual steps, the program automatically segmented the bone boundary and then the areas of high-signal intensity within the bone by performing a thresholding and curve evolution process twice. These areas of high-signal intensity represented a probable BML. We then used two criteria to exclude false-positive regions and to define a BML: (1) the distance between a BML and the articular surface should be ≤10 mm [39–41] and (2) a BML should appear on >1 MR image. The program output the BML volume for 4 different regions: medial femur, lateral femur, medial tibia, and lateral tibia.

Our reader had good reliability (intra-tester ICC 3,1 model baseline BML = 0.86 to 0.97). The reader also had good reliability compared with previous readers in our team (inter-tester intraclass correlation coefficient; ICC 2,1 model: baseline BML = 0.75 to 0.93). A second reader (JBD) reviewed all images for quality control, assuring that bone segmentation remained consistent across knees and time.

### Effusion measurements

Two readers measured knee effusion volume on sagittal intermediate-weighted, turbo spin echo, fat-suppressed MR images using a semi-automated program. Since the MR images were acquired without contrast the volumetric measurement may have included knee effusion and synovitis; however, throughout this manuscript we will describe the outcome as knee effusion volume. The program required the reader to first mark the first and last slice of the MR sequence in which the tibia or femur are visible. The reader then marked the base of the patella and the distal attachment of the patellar ligament into the tibial tuberosity on a middle slice. Following this, the program automatically applied a threshold to segment the effusion volume (areas of high-signal intensity) within the entire knee. The user then reviewed each slice with the option to manually adjust the threshold or remove areas of high-signal intensity that were not effusion (e.g., subchondral bone cysts).

Two readers (JBD and FA) used the semi-automated software to quantify changes in MR-based knee effusion. Intra-tester reliability was good (ICC 3,1 model): reader 1 (JBD) baseline = 0.96; reader2 [FA] baseline = 0.84). Since inter-tester reliability was = 0.42, reader 1 conducted a quality review of all segmented images to ensure consistent results between readers, knees, and time. The final data was based on the reviewed segmentation results that were corrected by reader 1.

### Biospecimens collection

Participation in the OAI was contingent upon a baseline fasting blood draw. Participants were asked to fast for >8 h prior to their study visit. Trained staff performed baseline blood draws at least 1 h after the participant had gotten up in the morning.

Immediately after collection, study staff inverted the serum sample tubes and kept them at room temperature for 30 min. Following these 30 min, the tubes were centrifuged at 4 °C for a total of 30,000 g-minutes and then immediately transferred to cryovials. Within 15 min of being transferred to cryovials the samples were placed in a − 70 °C freezer for at least 30 min prior to being shipped to Fisher Bioservices for long-term storage. In September 2015, Fisher Bioservices shipped the samples to Temple University School of Medicine. Additional details regarding the collection of biospecimens can be found in the OAI protocols, which are available on the OAI website [31].

### Biospecimens assay

All assays were performed at Temple University School of Medicine within 2 months of delivery. The samples were de-identified to ensure the laboratory staff was blinded. To assess serum high-sensitivity CRP, an enzyme-linked immunosorbent assay was used (ELISA, Novex by Life Technology, Carlsbad, CA; range = 18.75–1200 pg/mL, sensitivity = 18.75 pg/mL, coefficient of variation <7.52% for intra-assay precision, and coefficient of variation <10% for inter-assay precision). To assay GSP, an ELISA kit was used (MyBioSource, San Diego, CA; range = 78.125 μM – 1000 nmol/mL, sensitivity = 48.875 μM/mL, and coefficient of variation <10%). Samples used for glucose analyses were deproteinized prior to analysis with 10kD Spin Columns (Abcam, Cambridge, MA), per manufacturer’s directions. For fasting glucose levels, Glucose Assay Kits were used (Abcam; range = 1–1000 μM, sensitivity = 1 μM/mL, and coefficient of variation <2%). All samples were tested in duplicate.

### Other clinical variables

We extracted potential confounders from the public OAI data files: age, sex, body mass index (BMI), and Physical Activity Scale for the Elderly (PASE) scores (Files: enrollees, version 22; allclinical00, version 0.2.2). We also extracted self-reported diabetes from the Charlson Comorbidity Index. All variables were collected based on standardized procedures, which are defined in the OAI protocols, which are available on the OAI website (15).

### Statistical analyses

Initially, we calculated descriptive statistics; explored the distribution of CRP, GSP, and glucose concentrations, and applied the natural log (ln) transformation for GSP to stabilize the variable. Since our BML segmentation program detected small areas of increased signal intensity on every knee, we classified a knee as having a prevalent BML if any region (medial femur, medial tibia, lateral femur, lateral tibia) had a BML volume > 1 cm^3^. The BML cut-point was based on a prior classification and regression tree analysis with medial joint space narrowing progression as an outcome [37, 42]. A knee was classified as having prevalent effusion if the knee effusion volume was greater than the median knee effusion volume (> 7.5 cm^3^).

To assess the relationship between serum measurements (predictors) and BMLs or knee effusion (outcomes) we used logistic regression models, adjusting for age, sex, BMI, and PASE score. In separate models, we explored interactions between the biomarkers and BMI. We checked for a linear relationship between the three predictors and the log odds for the presence of BMLs or effusion using a plot spline transformation with linear hypothesis test (SAS macro: %PSPLINET, 3 knots). When a nonlinear relationship was present we performed piece-wise logistic regression analyses and selected the cut-point based on spline graphs.

We conducted 2 sensitivity analyses: 1) adjusting for self-reported smoking ever in life and 2) only including people who reported no diabetes. We also explored if there was an interaction between sex and any of the three serum measurements.

All analyses were performed with SAS Enterprise Guide 7.13 (Cary, NC).

## Results

We had complete data for 343 participants. The cohort had a mean age of 59 ± 9 years, BMI of 27.9 ± 4.4 kg/m^2^, PASE score of 171 ± 82, and 64% were female. Only 10 participants reported diabetes on the Charlson Comorbidity Index (7 had missing data). Overall, the sample had a mean (standard deviation) total BML volume 0.92 (1.48) cm^3^, effusion volume 9.09 (6.28) cm^3^, CRP 3.31 (1.21) mg/L, lnGSP 5.48 (0.72), and glucose 110.19 (26.52) mg/dL.

Only CRP concentrations were associated with the presence of BMLs (OR = 1.43, 95% CI = 1.09 to 1.87; Table 1). There were no associations between GSP or glucose levels and the presence of BMLs.

**Table 1:** C-reactive protein is associated with the prevalence of bone marrow lesions (BMLs)

Predictor (continuous)	No BML (*n* = 287) Mean (SD) (Reference)	Prevalent BML (*n* = 56) Mean (SD)	Prevalent BML Crude OR (95% CI), *p*-value (per unit of biomarker concentration)	Prevalent BML Adjusted OR (95% CI), *p*-value (per unit of biomarker concentration)
C-reactive Protein (mg/L)	3.24 (1.22)	3.67 (1.12)	**1.36 (1.05, 1.75), 0.02**	**1.43 (1.09, 1.87), 0.01**
Glycated serum protein (log)	5.50 (0.70)	5.43 (0.82)	0.87 (0.58, 1.32), 0.52	0.97 (0.65, 1.46), 0.90
Glucose (mg/dL) per 10 mg/dL	109.3 (27.2)	114.6 (22.2)	1.08 (0.97, 1.20), 0.18	1.07 (0.95, 1.19), 0.28

Adjusted analyses include age, body mass index, sex, and Physical Activity Scale for the Elderly Score. *OR* odds ratio, *95% CI* 95% confidence interval. Bold represents statistically significant findings (*p* < 0.05)

We found a statistical interaction between BMI and CRP when assessing effusion: among adults with a BMI <25 kg/m^2^, there was a trend for a positive association between CRP and knee effusion (OR = 1.40, 95% CI 1.00 to 1.97) but this association was null for overweight or obese individuals (OR = 0.83, 95% CI = 0.65 to 1.07). We detected a nonlinear relationship between lnGSP levels and the presence of baseline effusion: for lnGSP levels ≥5.5cm^3^, individuals with greater GSP concentrations were more likely to have baseline effusion (OR = 2.02). In contrast, for lnGSP levels <5.5cm^3^, individuals with lower GSP concentrations were more likely to have effusion (OR = 0.39; see Table 2). Fasting glucose levels were not significantly associated with the presence of baseline effusion.

**Table 2:** Glycated serum protein is associated with the prevalence of effusion

Predictor (continuous)	No-Little Effusion (*n* = 173) Mean (SD) (Reference)	Effusion (≥ 7.5 cm^3^) (*n* = 170) Mean (SD)	Prevalent Effusion Crude OR (95% CI), *p*-value (per unit of biomarker concentration)	Prevalent Effusion Adjusted OR (95% CI), *p*-value (per unit of biomarker concentration)
CRP (mg/L)	3.29 (1.27)	3.34 (1.16)	1.04 (0.87, 1.24), 0.68	1.01 (0.83, 1.22), 0.93
lnGSP <5.5	5.10 (0.29)	4.99 (0.40)	**0.36 (0.18, 0.73), 0.004**	**0.39 (0.18, 0.83), 0.01**
lnGSP ≥5.5	6.01 (0.49)	6.29 (0.71)	**2.01 (1.22, 3.32), 0.01**	**2.02 (1.22, 3.34), 0.01**
Glucose (mg/dL) per 10 mg/dL	108.7 (27.9)	111.7 (25.1)	1.04 (0.96, 1.13), 0.31	1.03 (0.95, 1.12), 0.48

All analyses were adjusted for age, body mass index, sex, and Physical Activity Scale for the Elderly score. *CRP* C-reactive Protein, *lnGSP* glycated serum protein (log), *OR* odds ratio, *95% CI* 95% confidence interval. Bold represents statistically significant findings (*p* < 0.05)

In sensitivity analyses, we found that adjusting for smoking or limiting the sample to people with no diabetes had little influence on the estimated odds ratios (< 10% change in OR). We found no significant interactions with sex.

## Discussion

Among adults without knee OA, we found that people with greater systemic inflammation (CRP concentrations) were more likely to have a BML. Furthermore, among individuals with a normal weight, adults with high CRP concentrations may be more likely to have knee effusion. Finally, GSP concentrations had a “U-shaped” relationship with effusion. These findings highlight the complex relationships that systemic inflammation and impaired glucose homeostasis may have among knees without OA.

Our results complement a prior study of people with knee OA in which the authors reported positive associations between quartiles of CRP and the prevalence and worsening of knee BMLs over 2 years [28]. We went beyond quartile readings and looked at CRP as a continuous variable, finding a linear relationship between CRP concentrations and BML presence. While Zhu, et al. [28] collected data from participants with symptomatic knee OA, our study focused on individuals without knee OA, but with established risk factors for the disease.

Only one study has looked at the relationship between glucose and BMLs. Specifically, the authors found a positive association between glucose levels and incident BMLs in women but not in men [30]; however, fasting glucose samples were collected >9 years prior to the initial MR images that were used for BML measurements. In contrast, we examined glucose concentrations in serum collected during the same visit as the MR images. Additionally, a novel aspect of our study was that we looked at a more stable metric of glucose homeostasis, GSP, rather than focusing purely on fasting glucose levels.

The inclusion of GSP in our analyses proved insightful as we found associations between GSP levels and the presence of baseline effusion. Our results complement our previous study that found a U-shaped correlation between GSP concentrations and incident knee OA: specifically, individuals with lnGSP concentrations <5.7 and individuals with lnGSP concentrations >5.7 were more likely to develop incident knee OA than those with lnGSP concentrations closer to 5.7 [32]. This U-shaped relationship may indicate that there are two clinically relevant subsets with effusion that warrant more study – those with low GSP concentrations and others with high GSP concentrations. Increased formation of advanced glycation end-products leads to increased collagen cross-linking, which can make the cartilage more brittle. This in turn can induce cartilage fragmentation [43], which could aggravate the synovium causing an increase in effusion volume [44, 45]. This may help explain the positive association between high GSP levels and effusion. Alternatively, the presence of knee effusion may lead to joint symptoms, which leads to a more sedentary life style and consequently a greater risk for impaired glucose homeostasis. Future studies should explore the cause for this association, as well as why low GSP levels are associated with effusion.

We also observed a trend that CRP had a positive association with effusion among individuals with a normal weight (BMI <25 kg/m^2^) but not among adults who were overweight or obese. This may indicate that other factors besides systemic inflammation contribute to effusion among individuals who are overweight or obese. The increased joint load in an overweight or obese individual can cause cartilage damage [46], and cartilage fragments may aggravate the synovium [41, 45]. It is important to note that the cross-sectional nature precludes us from determining if the presence of effusion – or other pathologies that contribute to effusion – may contribute to greater systemic inflammation among normal weight adults but not among individuals who are overweight and obese.

There were several potential limitations to our study. With our limited sample size, we may not have adequate power to explore more interactions, especially with the presence of nonlinear relationships. Despite this limitation, we detected nonlinear patterns and interactions that may be important to future research. It is vital that future studies anticipate non-linear relationships between glucose homeostasis and effusion. Secondly, our study did not consider the association between local inflammatory biomarkers and BMLs or effusion. We chose to focus solely on the whole-body burden of glucose and inflammatory homeostasis. There are many systemic and local factors that may contribute to changes in glucose and inflammatory homeostasis. Hence, we cannot infer causation; however, despite other possible causes for altered glucose and inflammatory homeostasis, BMLs, and effusion they are associated. Thirdly, the cross-sectional nature precludes us from making any causal inferences or disentangling whether systemic inflammation or glucose homeostasis leads the joint to be susceptible to BMLs or effusion or if the presence of BMLs and effusion may contribute to changes in systemic inflammation or glucose homeostasis. Finally, we used a sample that was enriched to include people who later develop radiographic knee OA and not a population-based sample, which would enable results that are more generalizable. Future population-based studies may build on these findings. Despite these limitations, we found novel associations and interactions that warrant further study.

## Conclusions

We found that systemic inflammation, specifically CRP, may be associated with BMLs and effusion in normal weight individuals. Furthermore, glucose homeostasis, determined using GSP, may be associated with effusion. Though our results indicate that inflammatory and glucose homeostasis are related to structural features of OA, it will be valuable to determine whether inflammatory and glucose homeostasis are related to incident symptomatic OA. Further understanding of these associations may help us develop strategies to prevent or delay symptomatic OA or alternatively to prevent an adverse influence of knee OA on systemic inflammation and impaired glucose homeostasis.

## Abbreviations

BMI: Body mass index
BML: Bone marrow lesion
CI: Confidence interval
CRP: C-reactive protein
GSP: Glycated serum protein
ICC: Intraclass correlation coefficient
MR: Magnetic resonance
OA: Osteoarthritis
OAI: Osteoarthritis Initiative
OR: Odds ratio
PASE: Physical Activity Scale for the Elderly
